# Glucocorticoids are lower at delivery in maternal, but not cord blood of obese pregnancies

**DOI:** 10.1038/s41598-017-10266-5

**Published:** 2017-08-31

**Authors:** Laura I. Stirrat, George Just, Natalie Z. M. Homer, Ruth Andrew, Jane E. Norman, Rebecca M. Reynolds

**Affiliations:** 1 Tommy’s Centre for Maternal and Fetal Health, MRC Centre for Reproductive Health, University of Edinburgh, Edinburgh, UK; 2 Mass Spectrometry Core, Edinburgh Clinical Research Facility, University of Edinburgh, Edinburgh, UK; 3 University/BHF Centre for Cardiovascular Science, University of Edinburgh, Edinburgh, UK

## Abstract

Glucocorticoids are vital for lung maturation. We previously showed that cortisol is lower in obese pregnancy. Whether this is maintained at delivery is unknown but is clinically relevant as maternal and cord blood cortisol levels are correlated and offspring of obese are more likely to need neonatal respiratory support. We hypothesized that glucocorticoids are lower in maternal and cord blood at delivery in obese pregnancies. Glucocorticoids (cortisol and corticosterone) and their inactive versions (cortisone and 11-dehydrocorticosterone) were measured by LC-MS/MS in maternal and cord plasma from 259 Caucasian women at delivery (BMI 18–55 kg/m^2^). Analyses adjusted for labour status, delivery mode, offspring gender, birthweight and gestational age. Cortisol and corticosterone were significantly higher in maternal than cord blood. Inactive versions were significantly higher in cord than maternal blood. Increased maternal BMI associated with lower maternal cortisol, corticosterone and 11-dehydrocorticosterone. Despite significant positive correlations between maternal and cord blood glucocorticoid levels, increased maternal BMI was not associated with lower cord blood glucocorticoid levels. Conditions at delivery may overcome any potential negative effects of low maternal glucocorticoids on the fetus in the short-term. This may not preclude the longer-term effects of fetal exposure to lower glucocorticoid levels during obese pregnancy.

## Introduction

Glucocorticoids are vital for fetal growth and lung maturation. This has been demonstrated therapeutically when glucocorticoids are administered antenatally to women at threat of preterm labour with consequent reduced respiratory morbidity in both extremely preterm^1^, and nearer term babies^2^. We and others have shown that circulating maternal cortisol levels are lower in obese compared with lean women during pregnancy^3–5^ and postpartum^3^. Whether or not lower maternal cortisol levels in obese are maintained at delivery is clinically relevant as maternal and cord blood cortisol levels are correlated^6–8^. As offspring born to obese women are more likely to need respiratory support at delivery^9^, it is plausible that exposure to comparatively lower levels of glucocorticoids *in utero* could limit fetal lung maturation in this group. One previous study reported lower maternal cortisol, but not cord cortisol at delivery in obese compared to normal weight women^4^, but did not adjust for mode of delivery or labour; both factors are known to influence cord cortisol levels^10–14^.

While cortisol is the major circulating glucocorticoid hormone in humans, there is increasing interest in the potential physiological roles of corticosterone, which comprises 5–10% of total plasma glucocorticoids^15^. The observation of proportionally greater increases in cord blood corticosterone than cortisol according to ‘stressful’ labour and mode of delivery has led to the suggestion that the full-term human fetus preferentially secretes corticosterone in response to fetal stress^14^.

To our knowledge there are no studies measuring maternal corticosterone levels at time of delivery and it is not known whether corticosterone levels differ in obese and lean pregnancy. Both cortisol and corticosterone can freely cross the placenta from mother to fetus, and are metabolised to their inactive forms (cortisone and 11-dehydrocorticosterone, respectively) by the placental enzyme 11beta-hydroxysteroid dehydrogenase type 2 (11β-HSD2).

We hypothesized that cortisol and corticosterone levels measured at the time of delivery would be lower in the maternal and cord blood of obese pregnancies than lean, even after adjusting for potentially confounding factors such as labour and mode of delivery. We tested this hypothesis by testing associations of maternal BMI with cortisol, corticosterone and their metabolites, measured in maternal and cord blood samples obtained at delivery from women across a range of obesity levels.

## Method

### Clinical Methods/Participants

We selected matched maternal and cord blood samples of 259 pregnancies from the Edinburgh Reproductive Tissue BioBank (ERTBB; ethical approval REC09/S0704/3), collected between January 2010 – December 2014. The ERTBB stores anonymised tissue specifically collected for pregnancy research with the majority of samples collected at time of elective Caesarean section. Tissue samples are linked to a database containing clinical records. Our sample set comprised 102 lean (BMI 18.5–24.9 kg/m^2^), 79 overweight (BMI 25.1–29.9 kg/m^2^), 45 obese (BMI 30–39.0 kg/m^2^) and 33 severely obese (BMI ≥ 40 kg/m^2^) women. Eligible women were Caucasian, had a singleton pregnancy, a normal booking ultrasound scan, and had not received any glucocorticoid therapy during their pregnancy. Clinical outcomes were extracted from clinical records. Macrosomia was defined as birthweight ≥4000 g at term (≥37 weeks).

### Biological Samples

Trained research midwives and research technicians collected maternal and umbilical cord plasma at the time of delivery. Samples were collected in chilled EDTA vials and centrifuged within one hour of collection. Plasma was separated and stored at −80 °C until analysis.

### Laboratory methods

*Plasma steroid extraction and LC-MS/MS quantification:* A method was developed to measure cortisol, cortisone, corticosterone and 11-dehydrocorticosterone simultaneously by liquid chromatography tandem mass spectrometry (LC-MS/MS), using an ABSciex QTRAP® 5500 (Warrington, UK) operated in positive ion electrospray ionisation, with a Waters Acquity™ UPLC system (Manchester, UK). Analytes were extracted from plasma (200 µL) via liquid-liquid extraction (chloroform 10:1 (v/v)) with epi-cortisol (25 ng; Steraloids, USA), epi-corticosterone (25 ng; Steraloids, USA) and 9,11,12,12 [^2^H_4_] cortisol (d_4_-cortisol; 25 ng: QMX Laboratories, UK) included as internal standards. Analytes were separated at 40 °C on a Waters Sunfire^TM^ C18 (2.1 × 150 mm; 3.5 μm) column (Manchester, UK) using an isocratic solvent system (70:30 of water with 0.1% formic acid and acetonitrile with 0.1% formic acid) with a gradient run of 7.1 minutes. Mass spectral conditions are demonstrated in Supplementary Table 1. Inter-assay precision and accuracy were within acceptable limits (Supplementary Table 3). The ratio of cortisol: cortisone was examined as a marker of 11β-HSD2 enzyme activity.

### Statistical analysis

Data distribution was assessed for normality by visually assessing histograms. Data that were not normally distributed were normalized using the natural-log transformation. The independent t-test was used to test for differences between continuous variables and chi-squared test for categorical variables. The one-way ANOVA was used to compare change in hormone levels between different groups. In regression analysis we adjusted for covariates and confounding factors known to influence glucocorticoid levels including mode of delivery or labour^16, 17^, and gestational age^18, 19^, or that differed between groups in our sample such as offspring birthweight. Model 1 adjusted for gestational age at delivery, offspring birthweight and mode of delivery. Model 2 adjusted for gestational age at delivery, offspring birthweight and labour status (labour or non-labour). Analysis was performed using SPSS v21 (IBM). Data in text are mean ± sd, and data in figures are mean ± SEM. Statistical significance was considered at p < 0.05.

## Results

### Demographics

Maternal and neonatal characteristics are demonstrated in Table 1. Obese and severely obese women were younger than lean women. Severely obese women had the highest numbers of current smokers. Women were well matched for parity, mode of delivery, gestational age at delivery and offspring gender. The most common mode of delivery in all BMI groups was elective Caesarean section, representative of samples in the ERTBB Induction of labour was highest in the severely obese group. Offspring birthweight was highest in the obese and severely obese groups. Lean women had significantly lower birthweight than both obese and severely obese women offspring. Rates of macrosomia were highest in the obese and severely obese group.

**Table 1:** Maternal and Neonatal Characteristics.

	Lean (n = 102)	Overweight (n = 79)	Obese (n = 45)	Severely Obese (n = 33)	p-value
**Maternal age** (years)	34.2 (5.0)	34.6 (6.2)	32.0 (5.2)	31.7 (5.0)	0.017
**Maternal BMI** (kg/m^2^)	22.6 (1.6)	27.3 (1.4)	34.2 (2.8)	44.3 (3.6)	<0.0001
**Parity** n (%)					
Prim	27 (26.5)	13 (16.5)	8 (18.8)	8 (24.2)	0.15
Para 1	50 (49.0)	48 (60.8)	26 (57.8)	12 (36.4)	
Para ≥ 2	25 (24.5)	18 (22.7)	11 (24.4)	13 (39.4)	
**Smoking** n (%)					<0.0001
Non smoker	96 (94.1)	75 (94.9)	44 (97.8)	22 (66.7)	
Smoker	6 (5.9)	4 (5.1)	1 (2.2)	11 (33.3)	
**Labour onset** n (%)					
Spontaneous	3 (2.9)	3 (3.8)	3 (6.7)	2 (6.1)	0.019
Induced	2 (2.0)	0 (0)	1 (2.2)	4 (12.1)	
Prelabour CS	97 (95.1)	76 (96.2)	41 (91.1)	27 (81.8)	
**Labour Type** n (%)					
Labour	9 (8.8)	3 (3.8)	3 (6.7)	5 (15.2)	0.212
Non-labour	93 (91.2)	76 (96.2)	42 (93.3)	28 (84.8)	
**Mode of Delivery** n (%)					
ElCS	93 (91.2)	76 (96.2)	42 (93.3)	28 (84.8)	0.102
SVD	0	0	0	1 (3.0)	
Instrumental	2 (2.0)	0	1 (2.2)	2 (6.0)	
EmCS in labour	3 (2.9)	3 (3.8)	2 (4.4)	2 (6.0)	
Prelabour EmCS	4 (3.9)	0	0	0	
**Gestational age at delivery** (days)					
Mean (sd)	273.7 (8.1)	275.3 (7.0)	275.4 (8.5)	276.3 (7.6)	0.285
**Baby gender** n (%)					
Male	55 (53.4)	40 (50.6)	27 (60)	20 (60.1)	0.607
Female	47 (46.1)	39 (49.4)	18 (40)	12 (36.4)	
**Birthweight** (g)					<0.0001
Mean (sd)	3442 (492)	3514 (546)	3851 (642)	3739 (555)	
**Macrosomia** n (%)					<0.0001
Yes	12 (11.8%)	11 (13.9)	20 (44.4%)	9 (26.5%)	

Parity was defined as ‘prim’ (no previous pregnancies delivered after 24 weeks), ‘para 1’ (one previous delivery after 24 weeks gestation) and ‘para ≥ 2’ (two or more previous deliveries after 24 weeks gestation). ‘Smokers’ were defined as those who considered themselves as current smokers. Macrosomia was defined as ≥ 4000 g. Data are mean (sd) or N (%). P-value is from one-way ANOVA for continuous variables, and chi-squared test for categorical variables.

**Missing data**: n = 1 baby gender missing from offspring of severely obese

**Key:** BMI (body mass index), CS (caesarean section), ElCS (elective caesarean section), EmCS (Emergency caesarean section), SVD (spontaneous vaginal delivery).

### Relationship between maternal and cord hormone levels

**Figure 1:**
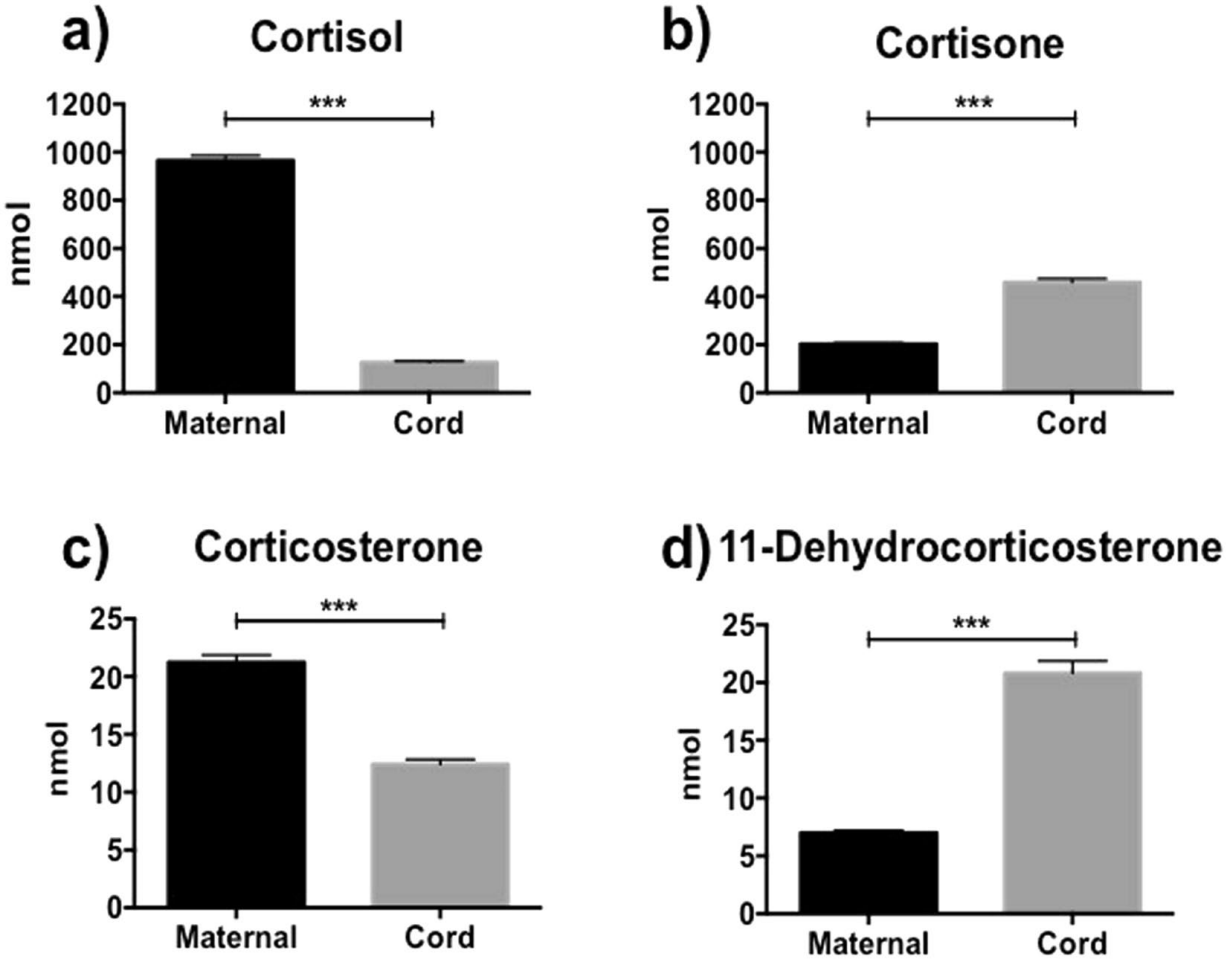
(**a**–**d**) Maternal and cord plasma levels. Cortisol (**a**), cortisone (**b**), corticosterone (**c**) and 11-dehydrocorticosterone (**d**). Data are mean (sem); ***p < 0.0001.

**Figure 2:**
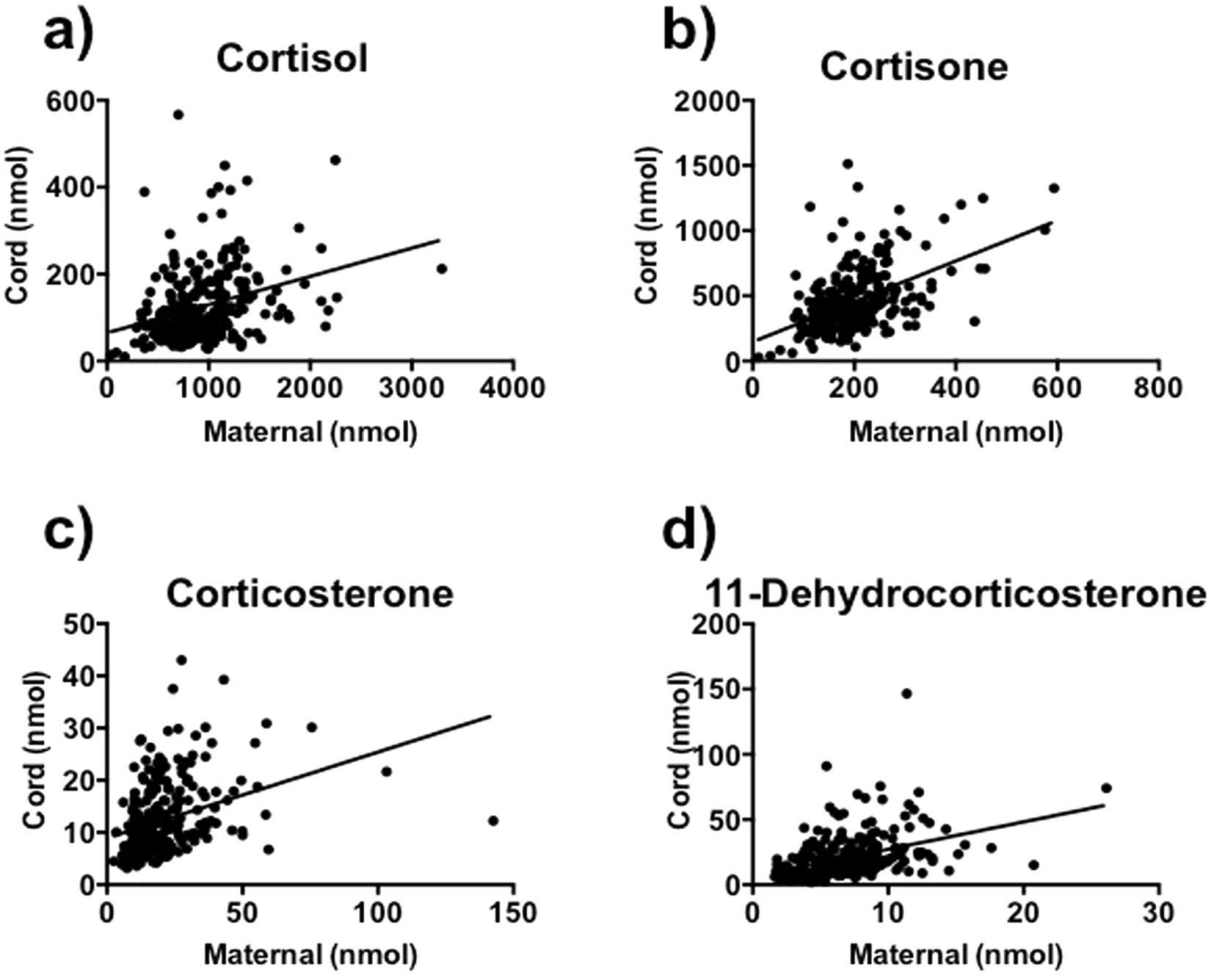
(**a**–**d**) Correlations of maternal and cord blood levels. Cortisol (r = 0.392, p < 0.0001; **a**), cortisone (r = 0.432, p < 0.0001; **b**), corticosterone (r = 0.437, p < 0.0001; **c**), 11-dehydrocorticosterone (r = 0.481, p < 0.0001; **d**).

Levels of cortisol and corticosterone were significantly higher in maternal blood than cord blood, whereas cortisone and 11-dehydrocorticosterone were significantly higher in cord blood than maternal blood (Fig. 1). Maternal and cord blood levels of all the monitored steroid hormones were significantly positively correlated (Fig. 2). Maternal cortisol and corticosterone were also positively correlated with cord blood cortisone and 11-dehydrocorticosterone levels respectively (r = 0.454, p < 0.001; r = 0.437, p < 0.001).

### Predictors of glucocorticoid levels at delivery

None of the hormones differed according to parity, maternal smoking or maternal age. Maternal corticosterone was significantly higher in labouring than non-labouring deliveries and was associated with greater ‘stress’ (vaginal delivery higher than emergency and elective Caesarean sections) (Table 2). Cord corticosterone was significantly higher in labouring than non-labouring deliveries but did not differ with mode of delivery. Cord cortisone was significantly higher in labouring than non-labouring, and was significantly higher in deliveries associated with greater ‘stress’ (vaginal delivery higher than elective Caesarean section).

**Table 2:** Hormone levels by labour status and mode of delivery.

Blood Type	Hormone	Labour Status			Mode of Delivery			
		Labour (n = 16)	Non-labour (n = 243)	p-value	Elective CS (n = 239)	Emergency CS (n = 14)	Vaginal delivery (n = 6)	p-value
Maternal Blood	11-dehydrocorticosterone	7.7 (3.8)	6.9 (3.1)	0.59	6.9 (3.1)	6.8 (3.4)	9.9 (4.2)	0.099
	Corticosterone	34.1 (30.2)	20.1 (11.6)	0.006	20.1 (11.6)	24.0 (16.3)	57.4 (43.0)	<0.0001
	Cortisone	198.7 (77.8)	202.5 (77.4)	0.857	202.1 (77.4)	184.9 (57.9)	230.9 (111.7)	0.637
	Cortisol	1086.3 (558.4)	928.7 (398.0)	0.293	928.7 (398.0)	1026.6 (597.2)	1225.7 (473.5)	0.266
Cord Blood	11-dehydrocorticosterone	26.3 (19.7)	20.4 (16.5)	0.076	20.4 (16.5)	22.4 (15.1)	35.5 (27.3)	0.072
	Corticosterone	15.5 (7.6)	12.2 (6.8)	0.046	12.2 (6.8)	14.2 (7.4)	18.4 (8.0)	0.058
	Cortisone	661.2 (342.5)	442.8 (226.6)	0.002	442.8 (226.6)	612.7 (332.9)	774.4 (368.3)	0.004
	Cortisol	145.7 (100.1)	124.4 (83.0)	0.35	124.4 (83.0)	140.1 (114.9)	158.9 (58.6)	0.364

Data are mean (sd). P-value from Student’s t-test for labour status, and from One-way ANOVA for mode of delivery.

The association of maternal BMI with glucocorticoid hormone levels is demonstrated in Table 3. In the unadjusted analysis, increased maternal BMI was associated with lower maternal cortisol, corticosterone and 11-dehydrocorticosterone. Maternal BMI remained significant as an independent predictor of these hormones in the adjusted analyses (Table 3). In cord blood, there were no associations between maternal BMI and glucocorticoid levels in the unadjusted or adjusted analyses.

**Table 3:** Associations of maternal BMI with hormone levels.

Blood Type	Steroid Hormone	Unadjusted		Model 1		Model 2	
		β 95% CI	p	β_a_ 95% CI	p_a_	β_a_ 95% CI	p_a_
Maternal Blood	11-DHC	−0.011 (−0.019 to −0.004)	0.003	−0.014 (−0.022 to −0.007)	< 0.0001	−0.014 (−0.022 to −0.007)	<0.0001
	Corticosterone	−0.010 (−0.019 to −0.002)	0.019	−0.12 (−0.021 to −0.004)	0.006	−0.013 (−0.022 to −0.004)	0.004
	Cortisone	0.0000052 (−0.007 to 0.007)	0.999	−0.003 (−0.009 to 0.004)	0.442	−0.003 (−0.009 to 0.004)	0.448
	Cortisol	−0.009 (−0.017 to −0.001)	0.023	−0.011 (−0.019 to −0.003)	0.009	−0.011 (−0.019 to −0.003)	0.007
Cord Blood	11-DHC	0.004 (−0.007 to 0.016)	0.472	0.005 (−0.007 to 0.016)	0.711	0.004 (−0.008 to 0.016)	0.358
	Corticosterone	0.000058 (−0.008 to −0.009)	0.989	−0.001 (−0.010 to 0.007)	0.739	−0.002 (−0.011 to 0.007)	0.672
	Cortisone	0.006 (−0.003 to 0.016)	0.168	0.005 (−0.004 to 0.014)	0.306	0.004 (−0.005 to 0.014)	0.377
	Cortisol	0.005 (−0.005 to 0.016)	0.303	0.002 (−0.009 to 0.012)	0.769	0.001 (−0.009 to 0.012)	0.800

Model 1: adjusted for gestational age at delivery, offspring birthweight and mode of delivery. Model 2: adjusted for gestational age at delivery, offspring birthweight and labour status (labour or non-labour).

## Discussion

Our findings demonstrate that increased maternal BMI was associated with lower maternal cortisol, corticosterone and 11-dehydrocorticosterone measured at time of delivery. Despite significant positive correlations between maternal and cord blood hormone levels, maternal BMI was not associated with cord blood glucocorticoid levels.

Our observation that cortisol and corticosterone were higher in maternal than cord blood, and that cortisone and 11-dehydrocorticosterone were higher in cord than maternal blood supports the hypothesis that the placenta acts as a barrier to protect the fetus from overexposure to active glucocorticoids^20^. Indeed, maternal cortisol and corticosterone were positively correlated with cortisone and 11-dehydrocorticosterone respectively. We also found a significant positive correlation between maternal and cord hormone levels (for all hormones), consistent with studies showing that maternal plasma and amniotic fluid cortisol levels are correlated during pregnancy^6–8, 21^.

Our findings are consistent with literature showing that labour and mode of delivery are associated with both a maternal and fetal endocrine (glucocorticoid hormone) stress response. For example, both higher maternal and cord blood cortisol levels have been reported where delivery was by vaginal or emergency Caesarean section (i.e. where labour had occurred), compared with delivery by elective Caesarean section (i.e. pre-labour)^10, 12, 14^. Our finding of higher corticosterone (but not cortisol) in labouring cord samples was in-keeping with a previous study^14^ who found highest corticosterone levels in vaginal deliveries, followed by emergency caesarean deliveries (who would have laboured), and lowest levels in elective caesarean deliveries (no labour). It has been suggested that this may be due to developmental changes in fetal glucocorticoid synthesis with preferential secretion of corticosterone in utero, and a shift towards the adult pattern of preferential cortisol synthesis after delivery^14^. These observations suggest that fetal adrenal corticosterone may be a better marker of fetal stress^14^. In addition, we showed for the first time that maternal corticosterone levels were higher in labouring than non-labouring women, which may suggest this hormone is released into the maternal circulation in response to the ‘stress’ associated with labour. However, we did not replicate previous findings of higher cord cortisol in cases where labour has occurred^10–12^; a large proportion of our subjects were delivered by elective Caesarean section, and more labouring samples may be required to replicate this finding.

Smith *et al*.,^11^ found no effect of maternal mood or anxiety disorders on cord blood cortisol at delivery, and suggested that conditions at delivery *per se* overwhelm the possible smaller diagnosis of treatment-related differences in hypothalamic-pituitary-adrenal axis responses during pregnancy. However, they did not adjust for mode of delivery or labour. Our data suggest that even in the ‘controlled’ conditions of elective Caesarean section, in accord with these findings^11^ the conditions at delivery *per se* may overwhelm the effects of BMI on cord cortisol. While detailed studies conducted at elective Caesarean section have informed our knowledge of maternal, placental and fetal glucose transfer^22^ such studies are less likely to be informative about glucocorticoid transfer between the maternal, placental and fetal unit.

A novelty of our study is the measurement of maternal corticosterone levels. In rodents, corticosterone is the major circulating glucocorticoid hormone and a handful of studies have measured maternal and fetal glucocorticoid levels in animal models of obesity in pregnancy. Two studies in mice and rats fed a high-fat diet reported increased levels of maternal corticosterone^23, 24^ suggesting that a high-fat diet acts as a stressful challenge during rodent pregnancy. However, in contrast to both of these studies, another study in rats measured rhythmic 21-hour profiles of maternal and fetal corticosterone and 11-dehydrocorticosterone, and found that these were unaffected by obesity^25^. Whilst rodent studies can more easily control for complications of labour and delivery than human studies and so are important comparisons for the effect of obesity on glucocorticoid levels, the species in maternal glucocorticoid responses to obesity/high fat diet need to be considered in interpreting the observations.

Strengths of our study are the large sample size, with a high proportion of elective caesarean deliveries, and a wide range of maternal BMI. Our finding of higher birthweight in offspring of obese women is representative of what is expected in this sub-population of pregnant women^26^. Our samples were analysed by liquid chromatography tandem mass spectrometry, which is the gold standard for measuring glucocorticoid hormones, and facilitated the simultaneous analysis of multiple hormones from a small sample volume (200 uL). We are also the first to describe the relationship of all these glucocorticoid hormones between maternal and cord blood.

A limitation of this study is that, like others^11, 12^ our cord blood samples were mixed cord artery and vein meaning we were unable to conduct a detailed assessment of the placental and fetal contribution to corticosterone metabolism. This was due to limited availability of cord blood in our setting, where delayed cord clamping is practiced and clinical bloods are required for blood gas testing; thus, the volume of blood available for research purposes was limited. Some studies have demonstrated differences in levels of cortisol and corticosterone between cord artery and vein^10, 14^. For example, in a study of 256 matched arterial and venous cord blood samples, Wynne-Edwards *et al*., reported an increase in both cortisol and corticosterone from the venous to the arterial circulation, suggesting that the fetal adrenal contributed corticosteroids to the arterial circulation while placental 11β-HSD2 was clearing both corticosteroids^14^. *Ex vivo* studies using the placental perfusion model^20^ combined with deuterated cortisol tracers^27^ would allow more details assessment of the fetal and placental contribution to corticosteroid levels.

In conclusion, our study suggests that maternal glucocorticoids are lower at delivery in obese pregnancy, and that conditions at delivery may overwhelm any effect of BMI on cord glucocorticoid levels. This should be considered in studies investigating glucocorticoids at delivery. Though conditions at delivery may overcome any potential negative effects of low glucocorticoids on the fetus in the short-term, this may not preclude the longer-term effects of lower glucocorticoid exposure during obese pregnancy and further studies are needed to examine this.

## Electronic supplementary material

Supplementary Tables
