# Supplementary material for: Glucocorticoids are lower at delivery in maternal, but not cord blood of obese pregnancies
Source: Sci Rep. 2017 Aug 31;7:10263. doi: 10.1038/s41598-017-10266-5 (PMC5579291; doi:10.1038/s41598-017-10266-5)
Supplement: Supplementary file 1 — Supplementary Tables [file 41598_2017_10266_MOESM1_ESM.doc]

**Glucocorticoids are lower at delivery in maternal, but not cord blood of obese pregnancies**

**Authors:** Laura I Stirrat^1^, George Just^2^, Natalie ZM Homer^2^, Ruth Andrew^2,3^, Jane E Norman^1^, Rebecca M Reynolds^1,3^

1. Tommy’s Centre for Maternal and Fetal Health, MRC Centre for Reproductive Health, University of Edinburgh

2. Mass Spectrometry Core, Edinburgh Clinical Research Facility, University of Edinburgh

3. University/BHF Centre for Cardiovascular Science, University of Edinburgh

# Supplementary Table 1 Mass spectral conditions for analysis of steroid hormones and internal standards using positive ion electrospray ionisation.

Key : Da (Daltons), Quan (quantifier ion), Qual (qualifier ion), V (volts)

|  | Molecular Weight (Da) | Precursor ion (*m*/*z*) | Product ion  Quan; Qual | Declustering potential  (V) | Collision energy  (V)  Quan; Qual | Cell exit potential  (V)  Quan; Qual |
| --- | --- | --- | --- | --- | --- | --- |
| **ANALYTES** | | | | | | |
| Cortisol | 362.5 | 363.2 | 121, 77 | 131 | 29, 101 | 14, 14 |
| Cortisone | 360.4 | 361.2 | 77; 163.2 | 166 | 99; 11 | 36; 20 |
| Corticosterone | 346.5 | 347.1 | 121.1; 97.1 | 66 | 69; 69 | 8; 8 |
| 11-dehydrocorticosterone | 344.4 | 345.1 | 121.2; 90.9 | 51 | 33; 71 | 8; 10 |
| **INTERNAL STANDARDS** | | | | | | |
| D_4_-cortisol | 366.5 | 367.0 | 121; only one | 121 | 25 | 20 |
| Epi-cortisol | 362.5 | 363.2 | 121; only one | 131 | 29 | 14 |
| Epi-corticosterone | 346.5 | 347.1 | 121; only one | 66 | 69 | 8 |

# Supplementary Table 2 Lower limits of quantitation

Key: LLOQ (lower limits of quantitation)

|  | Standard curve range  (ng/200 μL) | LLOQ  (ng/200 μL) |
| --- | --- | --- |
| Cortisol | 0.1 - 200 | 0.25 |
| Cortisone | 0.1 - 200 | 0.5 |
| Corticosterone | 0.1 - 200 | 0.25 |
| 11-dehydrocorticosterone | 0.1 - 200 | 0.25 |

# Supplementary Table 3 Inter-assay precision and accuracy

|  | **Target Concentration**  **(ng/200 μL)** | **Inter-assay (n=6)** | | |
| --- | --- | --- | --- | --- |
|  |  | **Concentration (ng/200 μL): mean (SD)** | **Precision**  **(% RSD)** | **Accuracy**  **(%)** |
| **Cortisol** | Low (0.25) | 0.27 (0.04) | 14.7 | 108 |
|  | Mid (50) | 53.7 (2.5) | 2.5 | 107 |
|  | High (200) | 178.3 (12.8) | 7.2 | 89 |
| **Cortisone** | Low (0.5) | 0.52 (0.1) | 18.7 | 105 |
|  | Mid (50) | 51.7 (3.1) | 6.0 | 103 |
|  | High (200) | 177.6 (12.3) | 6.9 | 89 |
| **Corticosterone** | Low (0.25) | 0.3 (0.05) | 15.5 | 99 |
|  | Mid (50) | 50.2 (3.2) | 6.3 | 100 |
|  | High (200) | 190.6 (6.8) | 3.6 | 95 |
| **11-dehydrocorticosterone** | Low (0.25) | 0.26 (0.03) | 13.0 | 105 |
|  | Mid (50) | 54.6 (52.0) | 3.4 | 104 |
|  | High (200) | 179 (20.0) | 11.2 | 89 |
